# Development and Characterization of Plantain (*Musa paradisiaca*) Flour-Based Biopolymer Films Reinforced with Plantain Fibers

**DOI:** 10.3390/polym14040748

**Published:** 2022-02-15

**Authors:** Ramiro Venegas, Andres Torres, Ana M. Rueda, Maria A. Morales, Mary J. Arias, Alicia Porras

**Affiliations:** 1 Grupo de Diseño de Productos y Procesos (GDPP), Department of Chemical and Food Engineering, Universidad de los Andes, CR 1 18a 12, Bogota 111711, Colombia; ra.venegas@uniandes.edu.co (R.V.); af.torres@uniandes.edu.co (A.T.); am.rueda13@uniandes.edu.co (A.M.R.); ma.morales12@uniandes.edu.co (M.A.M.); 2 Chemical Engineering Program, School of Engineering, Universidad Tecnológica de Bolívar, Parque Industrial y Tecnológico Carlos Vélez Pombo km 1 Vía Turbaco, Cartagena 130012, Colombia; mariast@utb.edu.co

**Keywords:** bio-based, plantain, natural fibers, agroindustrial waste, starch biopolymer

## Abstract

Agroindustrial wastes are a cheap and abundant source of natural fibers and macromolecules that can be used in the manufacturing of biocomposites. This study presents the development and thermo-mechanical characterization of a bio-composite film (TPF/PF), made of thermoplastic banana flour (TPF) matrix and plantain fibers (PF). Fabricated materials were characterized by physical analysis, chemical composition, Fourier-transformed spectroscopy (FTIR), thermal analysis (TGA), mechanical analysis, and scanning electronic microscopy (SEM). The physical analysis showed that TPF and PF have a low density and high affinity to water resulting in a lightweight, renewable, and biodegradable TPF/PF composite. The chemical composition and spectra analysis of the fiber showed that PF is a potential candidate for reinforcing composites due to its high α-cellulose and low lignin content. The thermal analysis determined that TPF degrades at a lower temperature than PF, therefore the matrix sets the processing temperature for TPF/PF composite films. The mechanical test showed an improvement in the tensile properties of the composite in comparison to neat TPF. Tensile strength and Young’s modulus were improved by 345% and 1196%, respectively, when PF fibers was used. Good bonding and mechanical interlocking of PF to the TPF were identified by SEM. Therefore, potential biocomposites can be developed using natural fibers and thermoplastic starches obtained from plantain agroindustrial wastes.

## 1. Introduction

Plastics are an essential part of modern economies and have been extensively used in a variety of fields, such as food packaging, electronics, aerospace, and more. However, the disposal of solid wastes in which plastics are included has proven to be a challenging endeavor. The issue of solid waste disposal has turned into an urgent environmental problem worldwide, especially petrol-derived plastics wastes. Therefore, governments and researchers worldwide have intensified their efforts to develop novel biocomposites in their quest to replace conventional plastic manufacturing with biocomposites [1].

Biocomposites are defined as biocompatible and/or eco-friendly composites, which can consist of organic polymers such as polysaccharides and proteins [2]. Such materials are important research topics in modern science and technology because they can help industries achieve a more carbon-neutral production and replace the use of non-renewable resources, like oil and gas, with renewable resources such as biomass [3]. Studies in the field of biocomposites have focused mainly on fibers such as hemp [4], flax [5], sisal [6], and bamboo [7]. Nevertheless, researchers have kept identifying new sources of potential natural fiber reinforcement that can improve the mechanical and physical properties of biocomposites, such as *Abelmoschus esculentus* [8], *Grewia tilifolia* [9], *Sterculia urens* [10], and *Prosopis juliflora* [11]. Following this trend, fibers extracted from the *Musa paradisiaca* (plantain) tree are highlighted as a viable composite reinforcement due to its favorable physical and mechanical characteristics [12]. Chamorro et al. [13] successfully tested the possibility of incorporating *Musa paradisiaca* fibers into thermosetting polymeric resins such as polyester and epoxy resins.

Exploring different composite matrices, organic biodegradable macromolecules, like proteins and polysaccharides, are becoming ever more attractive to researchers, due to their ability to form bio-based polymers. Even though macromolecules are found abundantly in most organic solid wastes in landfills and other trash disposal facilities, they continue to be left to decompose or even incinerate instead of using these wastes’ high potential as a source of bio-based polymers for the manufacturing industry [14]. Among these macromolecules, starch has been implemented in several studies [15,16,17,18] as the main component in the fabrication of biodegradable films and coating, indicating the high interest of researchers in implementing starch-based biopolymers, also known as thermoplastic starch, for product manufacturing. Its biodegradability, high commercial availability, and easy processing make starch very attractive for polymer production. Due to starch being mainly present in the edible part of the fruit, recent studies began to evaluate the possibility of using fruit-peel waste flour as a source of starch. Such flours contain not only starch but also proteins, lipids, fibers, and other polysaccharides [16,19,20]. The interest in using starch-based biopolymers, in combination with natural fiber reinforcements, lies in the possibility of creating added value products by using agroindustrial wastes.

The banana fruit and its varieties, such as plantain, are a renewable source from which both starch and natural fibers can be extracted. Unfortunately, it is used solely as a food crop, meaning that most of the residues of its life cycle (peels and pseudostem) have no current use in the industry [21], making finding new applications a research challenge. In particular, plantain as a source of starch for bio-based polymer production has been studied in the last decade. F.M. Pelissari et al. [19] compared the physical, thermal, mechanical, and morphological properties of both plantain starch-based and plantain flour-based polymer films, highlighting the potential of flour-based polymer films as a greener and cheaper alternative to the use of pure starch for manufacturing, as well as championing the tenets of a circular economy.

Even though the use of natural fibers as composite reinforcement and the development of bio-based matrices has been evaluated, the integration of natural fibers and starch bio-based matrices has not been deeply explored in literature. Therefore, the production of biocomposites combining plantain flour bio-based polymeric matrices and plantain natural fibers could combine both fields in the manufacturing of added-value products. This new approach in the development of biocomposites would assess the possibility of implementing most of the wastes of a single crop’s production (plantain fibers) and consumption (plantain peels) stages.

Hence, this work presents the development and characterization of a bio-composite film of plantain flour thermoplastic starch (TPF) reinforced with plantain tree fibers (PF). The chemical, thermal, mechanical, and morphological characterization of TPF, untreated PF, and the composite material (TPF/PF) are shown through chemical composition analysis, Fourier-transformed infrared analysis (FTIR), thermos-gravimetric analysis (TGA), tensile tests, and scanning electronic microscopy (SEM).

## 2. Materials and Methods

### 2.1. Materials

Unripe plantain bananas of the variety “Harton” were acquired during the 2021 harvest in local markets in Bogota, Colombia, and used to obtain the plantain flour. This flour is extracted to produce TPF, in combination with industrial-grade glycerin (87% *w*/*w*) provided by Roda Químicos S.A.S. Plantain fiber bundles with a diameter between 94–189 μm were provided by farmers from San Agustín, Huila, Colombia, to implement the fiber as reinforcement. This fiber was previously cut and combed manually by the providers.

### 2.2. Chemical Composition of Plantain Fibers

A chemical composition analysis of the plantain fiber was carried out following TAPPI methods. Ash, lignin, cellulose, and extractive contents were determined. A minimum of 3 samples were used in each test.

#### 2.2.1. Ash Content

This test was performed following the TAPPI T211 Standard Method [22]. For this procedure, a 1 g dry sample was exposed to a three-step drying cycle to avoid flames during the experiment. First, the sample was heated at 105 °C for 1 h, then heated at 250 °C for another hour, and finally at 575 °C for 4 h. This procedure guarantees good carbonization and the calcination of the sample. For this test, a VIBRA HT224R analytical scale and a Thermolyne 62700 furnace were used. The ash content was calculated as the recovered ash mass (m_ash_) divided by the initial sample mass (m_sample_), as explained in Equation (1).

(1) $$\mathrm{Ash}\;\mathrm{content}\;\left(\%\right)=\frac{m_{\mathrm{ash}}}{m_{\mathrm{sample}}}\times 100$$

#### 2.2.2. Lignin Content

This test was performed following the TAPPI T222 Standard Method [23]. A 1 g dry sample was treated with 72% (*w*/*w*) sulfuric acid for 2 h. The sample was then macerated and stirred using a glass rod to dissolve extractive contents entirely during this process. Next, water was added to the solution until a 3% sulfuric acid mixture was obtained. Afterward, the solution was heated for 4 h to maintain constant volume to get lignin to coagulate. Lignin content was filtered from the solution via glass vacuum filtration, washed with water at 90 °C, dried in a Memmert UNE 200 forced convection oven at 105 °C, cooled, and finally weighed. The lignin content was calculated as the ratio of recovered lignin mass (m_lig_) to the initial sample mass (m_sample_), as seen in Equation (2).

(2) $$\mathrm{Lignin}\;\mathrm{content}\;\left(\%\right)=\frac{m_{\mathrm{lig}}}{m_{\mathrm{sample}}}\times 100$$

#### 2.2.3. Cellulose Content

This test was performed following the TAPPI T203 Standard Method [24]. A 1.5 g sample was extracted using 17.5% sodium hydroxide solution at 25 °C. Alpha (α), beta (β), and gamma (γ) cellulose were determined via oxidation with potassium dichromate; therefore a two-step oxidation was carried out to determine the content of each cellulose type correctly. In the first oxidation, beta and gamma cellulose content was separated from α-cellulose content because α-cellulose is insoluble in sodium hydroxide. α-cellulose content was then determined as the difference between the initial specimen (100%) and the undissolved fraction, as seen in Equation (3).

(3) $$\alpha -\mathrm{cellulose}\;\mathrm{content}\;\left(\%\right)=100-\frac{6.85\times \left(V_{2}-V_{1}\right)\times N\times 20}{A\times W}$$

V_1_ corresponds to the sample titration volume; V_2_ corresponds to the blank titration volumen; N is the normality of the titrate (ferrous amonium); A is the volume of the sample used, and W is the dry weigth of the fiber sample. Subsequently, γ-cellulose was recovered in the second oxidation of the solution, leaving behind the β-cellulose precipitated content. γ-cellulose content was determined using Equation (4).

(4) $$\gamma -\mathrm{cellulose}\;\mathrm{content}\;\left(\%\right)=\frac{6.85\times \left(V_{4}-V_{3}\right)\times N\times 20}{25\times W}$$

where V_3_ corresponds to the sample titration volume after filtering the precipitated β-cellulose, and V_4_ corresponds to the blank titration volume. Finally, β-cellulose content was determined by the difference between the first and second oxidations, see Equation (5).

(5) β−cellulose content (%)=100−(α−cellulose+β−cellulose)

#### 2.2.4. Extractive Content

This test was performed following the TAPPI T204 Standard Method [25]. A 4 g sample was oven-dried at 105 °C and milled to obtain a 0.4 mm granule size. Using an ethanol-benzene mixture (1:2, *v*/*v*), the sample was boiled at 75 ± °C in a Soxhlet apparatus for 5 h. Afterward, the solvent mixture was evaporated, and the residue was weighed. Extractive content was calculated as the ratio of the extract dry weight (m_ext_) to the initial sample mass (m_sample_), as seen in Equation (6).

(6) $$\mathrm{Extractive}\;\mathrm{content}\;\left(\%\right)=\frac{m_{\mathrm{ext}}}{m_{\mathrm{sample}}}\times 100$$

### 2.3. Film and Composite Preparation

Plantain flour was extracted from plantain peels as a preliminary step to prepare the TPF films. Peels were cut into small pieces, and the endocarp was separated from the cellulose-rich outer peel. Then, the starch-rich endocarp was dried for 90 min at 105 °C and subsequently ground using a Pulversisette 19 Universal Cutting Mill to obtain a fine flour powder. Finally, the flour was sieved using a 100 µm sieve, achieving homogeneous particle sizes and removing unwanted cellulose residue.

Films were prepared by casting method [15,16,19], a process in which a film-forming suspension (FFS) is applied over a surface and dried until a film is obtained. The FFS was obtained by homogenizing an aqueous solution of 4% *w*/*w* flour employing mechanical stirring at a constant temperature between 70 °C and 80 °C for 30 min to dissolve the starch content of the flour adequately. Industrial-grade glycerin was added at 2% *w*/*w* to the solution and processed at the same conditions for 15 more minutes. The produced suspension was poured into a mold to obtain a constant thickness film. The suspension was dried in a Memmert UNE 200 forced convection oven at 50 °C for 1.5 h to achieve a fast initial plasticization. Finally, the molds were left to dry approximately for 72 h at 20 °C and 50% relative humidity. The final product is a film, which acts as the matrix for the composite.

The composite films were obtained using the casting method described previously. PF was placed and fixed in a unidirectional orientation on the mold. Approximately 60 individual fibers were placed at a separation distance of 1 mm to permeate the fibers thoroughly. FFS was added to the previously conditioned mold with the fibers in place and left to dry for approximately 72 h at 20 °C and 50% relative humidity. The resulting film contained 6.54% *w*/*w* fiber content.

### 2.4. Physical Characterization

The thickness of each specimen was measured using an Ono Sokki GS-332 Linear Sensor Gauge. To obtain an average thickness, five different measures, each 30.5 mm apart, were taken along the length of the specimen. The density of TPF and TPF/PF was determined as the ratio between the mass and volume. The moisture content of each specimen was determined by a Precisa XM 60 Thermobalance and then averaged, following the ASTM D4442 standard procedure [26]. A minimum of 10 samples of each material were used for this test.

### 2.5. Fourier-Transformed Infrared Spectroscopy (FTIR)

An FTIR test was carried out to analyze the functional groups present in the TPF, PF, and TPF/PF. For this test, all specimens were ground and mixed with KBr in a ratio of 100:1. The mixture was later homogenized and pressed at 9 MPa for 30 s to obtain a pellet. The wavelength range analyzed was between 4000 cm^−1^ to 400 cm^−1^. A Thermo Nicolet 380 FT-IR was used for these tests.

### 2.6. Thermo-Gravimetric Analysis (TGA)

Thermo-gravimetric analysis (TGA) tests for the TPF, PF, and TPF/PF, were carried out using a TA Instruments Q600 analyzer and following the ASTM E1131 [27] standard procedure. Samples were heated from room temperature (25 °C) to 600 °C at a rate of 10 °C/min in a nitrogen atmosphere.

### 2.7. Mechanical Characterization

Tensile properties of the TPF and TPF/PF were determined by an Instrom 3367 Universal Testing System following the D882-10 ASTM standard [28]. Rectangular specimens, each 25 mm wide and 152.4 mm long, were tested. The specimens were cut utilizing die-cutting. The initial crosshead speed and grip separation were set at 12.5 mm/min and 125 mm, respectively.

Additionally, tensile properties of PF were determined by an Instrom 3367 Universal Testing System according to the C1557 ASTM standard [29]. A 50 mm long gauge length with a 10 mm/min crosshead speed where used in this test.

A minimum of 10 specimens for each material were used for each test.

### 2.8. Scanning Electron Microscopy (SEM)

PF and the fractured surfaces of TPF/PF samples were observed by FE-MEB LYRA 3 TESCAN (SEM). Before the analysis, specimens were coated with a thin gold layer, increasing sample conductivity, using a Desk^®^ IV apparatus.

### 2.9. Statistical Analysis

An ANOVA one-way test was carried out to determine if the physical and mechanical properties of TPF and TPF/PF presented a significant difference. A *p*-value lower than 0.05 (confidence level of 95%) was considered statistically significant [30]. To perform the statistical analysis, Minitab 18 Statistical Software was used.

## 3. Results

### 3.1. Chemical Composition of Plantain Fibers

Natural fibers are composed mainly of cellulose and lignin with lesser amounts of other components such as ash and extractives. The composition of a natural fiber directly affects its physical and mechanical properties [31]; therefore, a chemical composition analysis of PF was carried out. As seen in Table 1, the main component is cellulose (81.05%), from which 69.09%, 0.36%, and 11.40% correspond to alpha (α), beta (β), and gamma (γ) cellulose, respectively. The main cellulose component in plantain fiber, α-cellulose, is an advantage for composites, because it has been reported to have high strength and stiffness per weight, allowing the formation of long fibrous cells [32]. Further, β-cellulose corresponds to degraded cellulose, and γ-cellulose is composed mainly of hemicellulose. In comparison to other natural fibers (see Table 2), PF shows a high cellulose content, making it a competitive reinforcement choice because it correlates to better mechanical performance [33,34]. In summary, PF’s high α-cellulose content makes it a suitable reinforcement for composite applications.

Similarly, as shown in Table 2, PF has a low/intermediate lignin content (13.17%), correlating to better fiber–matrix interface and increasing their compatibility [35]. This results in a more reliable adhesion to polymeric matrices when employed in composite manufacturing. Furthermore, the ash content (3.5%) and extractives content (2.28%) (see Table 1) are in the range of other natural fibers reported by other authors, 0.6–8% [36] and 0.8–14.3% [37], respectively. In conclusion, the chemical composition analysis displays that plantain fibers are a potential reinforcement alternative for the manufacturing of composite materials.

### 3.2. Physical Characterization

In composite engineering, the physical characteristics of the matrix and the reinforcement dictate the final properties of the composite; therefore, a physical characterization (moisture content and density) of TPF matrix, PF fiber, and TPF/PF composite was carried out. As seen in Table 3, the moisture content of TPF is 10.42%, this value is in the range of other plantain thermoplastic starch films found in literature, 6.50–15.24% [17,19,40]. This can be an advantage for composite applications because moisture creates a plasticizer effect, increasing the flexibility of the bio-based polymer [41]. Additionally, PF displays a low moisture content (8.39%) when compared to other widely used natural fibers, such as jute (12.00%), sisal (11.00%), coir (10.00%), and hemp (9.00%) [38]. When TPF and PF are implemented into a composite material, the result was a TPF/PF composite with an average moisture content of 9.90%. TPF/PF composite appears to have a high affinity to water molecules, due to the presence of hydrophilic components found in TPF and PF, such as starch, cellulose, and, in lower amounts, proteins. According to the ANOVA test, there was no significant difference in moisture content between TPF and TPF/PF (*p*-value = 0.051). In general, this hydrophilic composite behavior promotes its faster biodegradation at the end of its life cycle, which is an advantage when designing short life-cycle products.

Regarding density, TPF’s value is 1.14 g/cm^3^ (see Table 3) which is on par with other plantain starch and flour bio-based polymers [19], and lower than that of synthetic polymer resins such as epoxy (1.2 g/cm^3^ [42]), phenolic (1.4 g/cm^3^ [43]), and cyanate ester (1.2 g/cm^3^). In the case of the fiber, it has a density of 0.83 g/cm^3^, a value lower than other widespread natural fibers like hemp (1.48 g/cm^3^), flax (1.5 g/cm^3^), sisal (1.5 g/cm^3^), and coir (1.2 g/cm^3^) [38]. Finally, the composite displays a density of 1.10 g/cm^3^, a good alternative to be used for lightweight products applications. Nevertheless, the ANOVA test determined that no significant difference in the densities of TPF and TPF/PF was present (*p*-value = 0.070). In general, the physical properties of TPF and PF allowed the manufacturing of a lightweight composite material.

### 3.3. Fourier-Transformed Infrared Spectroscopy (FTIR)

**Figure 1:**
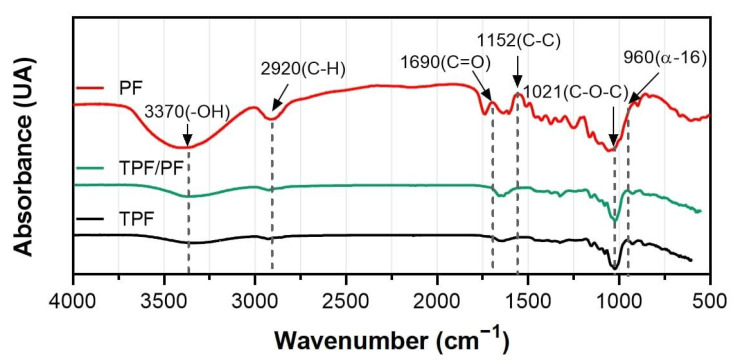
Fourier-transformed spectroscopy of the TPF, PF, and TPF/PF.

Figure 1 shows results from the FTIR spectra analysis for TPF, PF, and TPF/PF. Such analysis was conducted to have a better comprehension of the functional groups present in each material. TPF presents an energy absorption band around 3370 cm^−1^. These can be attributed to stretching –OH groups caused by the formation of hydrogen bonds [44]. Energy absorption bands around 2920 cm^−1^ are evidence of the presence of C–H and CH_2_ groups, most probably coming from amylose and amylopectin content [45], both are polysaccharides found in plantain flour. The peak around 1690 cm^−1^ corresponds to amide I, a functional group present mainly in proteins, in which the stretching of C=O groups occurs [20]. Other amide groups such as amide III are also present, correlating to the bands around 1323 cm^−1^ [46]. These amide groups are found mainly in organic proteins, meaning that plantain peel flour does contain protein material. Additional energy absorption bands around 1152 cm^−1^, 1077 cm^−1^, and 1021 cm^−1^ were associated with stretching the C–C, C–O, and C–O–H bonds of starch [47,48]. Finally, an energy absorption band was also observed around 960 cm^−1^, indicating the presence of glycosidic bonds in starch due to amylopectin α-16 bonds [45]. Even though different plantain varieties were used, the FTIR is in accordance with a similar analysis carried out by F.M Pelissari et al. [19] on plantain starch and flour polymeric films, as well as other starch-based films from other sources such as corn [49] and cassava [50]. In general, the main functional groups detected in TPF correspond to polysaccharides and glycosidic bonds which allow the formation of the polymeric matrix.

The previous chemical characterization revealed that the main components of PF were cellulose and lignin, substances also detected by the FTIR analysis. PF presents an energy absorption band around 3370 cm^−1^, confirming the presence of α-cellulose due to the stretching –OH groups caused by the formation of hydrogen bonds [44]. The peak around 2920 cm^−1^ correlates to the symmetric and asymmetric stretching of C–H and CH_2_ groups, also related to α-cellulose [32,51]. Bands around 1152 cm^−1^, 1077 cm^−1^, and 1021 cm^−1^, indicating the presence of asymmetrical stretching of C–C and C–O–C bonds also found in cellulose. Additionally, the peak around 1638 cm^−1^ corresponds to C=C bonds found primarily in lignin [51,52]. Finally, TPF/PF combines the behavior observed in both TPF and PF, the materials used as constituents of the composite.

### 3.4. Thermo-Gravimetric Analysis (TGA)

**Figure 2:**
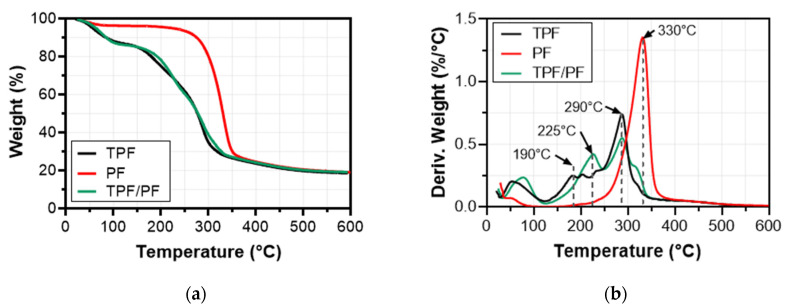
TGA (**a**) and DTGA (**b**) for TPF, PF, and TPF/PF.

The thermal stability of materials can determine processing conditions in composite applications; therefore, a thermo-gravimetric analysis was carried out for the TPF matrix, PF fiber, and TPF/PF composite. As seen in Figure 2, the matrix displays an initial phase, up to 120 °C, which is attributed to moisture evaporation [53,54,55]. A second phase takes place between 130 °C and 220 °C, due to the evaporation of a glycerol-rich phase. Finally, between 230 °C and 350 °C, a weight-loss step correlating to the degradation of the starch-rich phase is observed [56,57,58]. A residual mass percentage of around 17% was found at 600 °C. This thermal behavior agrees with other starch bio-based polymeric films reported by other authors in literature such as cassava starch films [52,55], sugar palm starch films [57], and corn starch films [58]. In summary, the thermo-gravimetric analysis determined that the degradation of TPF starts around 130 °C; therefore, it should be processed at temperatures below that.

Organic material in PF started to degrade at 240 °C, reaching a peak degradation temperature at 330 °C. Nevertheless, the degradation process of PF fibers extended well into the 500 °C range. This is attributed to the presence of lignocellulosic and inorganic substances found in the fiber [59]. These results show a similar thermal profile to that of other natural fibers such as okra (peak degradation at 335 °C), curaua (peak degradation at 350 °C), and jute (peak degradation at 283 °C), as well as other plantain thermal degradation analyses of other authors [60,61]. A 19% residual mass percentage was found at 600 °C. In brief, TGA determined that PF should be processed at temperatures below 240 °C to avoid fiber degradation.

TPF/PF shows thermal stability that combines its constituents’ thermal behavior. It exhibits an initial phase, up to 120 °C, representing the loss of moisture. This is followed by a second phase, between 130 °C and 220 °C, showing the loss of the glycerol-rich phase of the matrix and the early degradation of PF. Finally, the third phase from 230 °C onwards shows the degradation of both the starch-rich phase of the matrix (between 230 °C and 350 °C) and PF’s degradation process (between 240 °C up to 500 °C). The peak temperatures of the degradation of the starch-phase (290 °C) and PF (330 °C) can be found in the third phase. A residual mass percentage of 17% was found at 600 °C. In conclusion, the TPF/PF composite should not be exposed to temperatures above 130 °C to prevent the degradation of the matrix.

### 3.5. Mechanical Characterization

**Figure 3:**
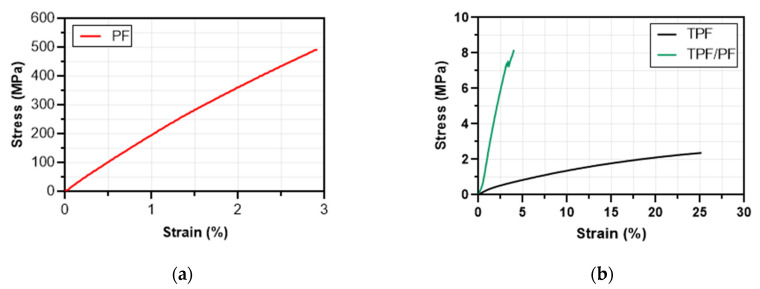
Stress vs. strain curves of PF (**a**), and TPF and TPF/PF (**b**).

As seen in Figure 3, all three materials show an initial linear region corresponding to an elastic behavior, followed by a plastic region characterized by a deviation from linearity until the final rupture of the material. Something to highlight in the TPF/PF curve is the steps present near the rupture of the sample. This behavior is attributed to the unidirectional configuration of the TPF/PF composite, in which fibers tend to break progressively until a total sample rupture is reached.

TPF films averaged a tensile strength and Young’s modulus of 2.36 MPa and 23.52 MPa, respectively (see Table 4). These results are comparable and, in some cases, higher than other starch and flour bio-based polymeric films such as batata starch and rice starch bio-based films (see Table 5). On the other hand, PF averaged a tensile strength and Young’s modulus of 440.24 MPa and 15.67 GPa, respectively. The fibers displayed a mechanical performance on par with other widely used natural fibers such as jute, sisal, and bagasse (see Table 6), meaning that plantain fibers could be used as reinforcement for a wide variety of polymeric matrices. Once PF fibers were incorporated into TPF films, the resulting TPF/PF composite averaged a tensile strength and Young’s modulus of 8.16 MPa and 281.34 MPa, respectively. This represents a 345.76% increase in tensile strength and an 1196% increase in Young’s modulus when compared to TPF films. In addition, specific properties of TPF/PF also increased by 518.84% for specific tensile strength and 1793.51% for specific modulus, when compared to TPF films (see Table 4). In conclusion, the TPF/PF composite films displayed a good mechanical performance. Its attractive properties show potential to be used, for example, as single-use flexible plastic packaging, where biodegradability, non-toxicity, and intermediate tensile properties are needed to achieve a circular-economy product.

### 3.6. Scanning Electron Microscopy (SEM)

**Figure 4:**
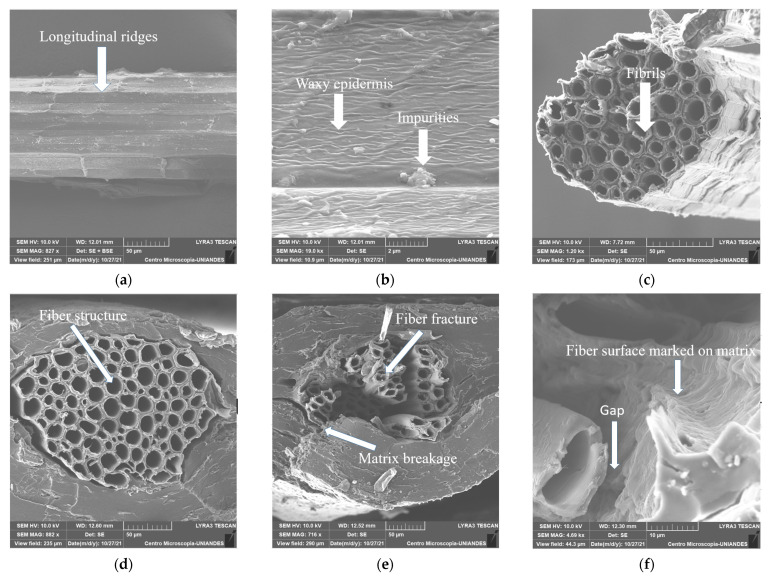
SEM images of PF at (**a**) ×827, (**b**) ×19,000, (**c**) ×1200 and TPF/PF (**d**) ×882, (**e**) ×716, (**f**) ×4690 longitudinal and cross sections.

SEM micrographs of PF fiber and tensile fractured TPF/PF composite were carried out to analyze their morphology and identify failure mechanisms in composite films. Figure 4a–c shows an overview of both longitudinal and cross-section of the fiber. The fiber was seen to have longitudinal ridges, impurities, and a waxy epidermis covering its surface. Moreover, the cross-section view shows that plantain fibers are organized as a bundle of round and hollow fibrils bonded together, a common structure for natural fibers. This binding of fibrils made possible by the effect of lignocellulosic intercellular material explains the low density of natural fibers [52].

With respect to TPF/PF, Figure 4d–f shows that the PF is completely embedded in the matrix, but its inner sections were not filled by resin. Fiber fracture occurred at different heights, due to the different mechanical performances of each fibril. Matrix fracture and fiber pull-out were also identified as failure mechanisms found on the specimens. The pulled fiber was indeed bonded to the polymeric matrix, as evidenced by the fiber surface marked on the matrix. This phenomenon indicates mechanical interlocking, which explains the improvement of the mechanical properties of the composite compared to those of the film. Nevertheless, fibers can also lack bonding with the polymeric matrix, resulting in gaps between them. This lack of fiber–matrix bonding is attributed to the waxy epidermis and impurities previously identified on the fiber’s surface [71]. In order to remove lignin waxes and impurities found on the surface, surface treatments such as mercerization and alkalization are some of the most common and compelling possibilities. These types of treatments have been shown to improve fiber/matrix adhesion and surface roughness, resulting in better mechanical interlocking [72,73,74]. In brief, the PF bonded successfully to the TPF films, but a better matrix/fiber interfacial interaction could be achieved by treating the fiber’s surface, resulting in better mechanical properties in TPF/PF.

## 4. Conclusions

In this study, a biocomposite from agroindustrial plantain waste with attractive mechanical properties and eco-friendly character was developed, contributing to the industrial conversion towards a circular economy.

The overall physical characterization shows that both the plantain flour-based biopolymer film (TPF) and plantain fibers (PF) are low-density and low-moisture materials when compared to synthetic polymer resins and other natural fibers, respectively. As a result, the TPF/PF composite has the advantage of being a lightweight, renewable and biodegradable material.

The chemical composition and spectral analysis of PF showed a high amount of α-cellulose and low amounts of lignin. These characteristics make PF a good candidate for reinforcing composite materials. In addition, the FTIR spectra of the TPF matrix and TPF/PF composite revealed the presence of OH and CH_2_ groups and amide I and III groups, which are found primarily in polysaccharides and proteins, respectively.

With regards to thermal degradation, it has been determined that, for TPF matrices, it starts at 130 °C, while PF fibers start degrading at 240 °C. The TPF/PF composite displayed the behavior of its constituent materials; therefore its processing temperature is set to below 130 °C to avoid unwanted degradation of the material.

The mechanical properties of both TPF and PF proved to be comparable to other starch bio-based resins and natural fibers, respectively. When combined into TPF/PF, composite films displayed a superior performance in comparison to TPF films, showing plantain fibers as effective reinforcement. SEM analysis showed a good bonding between TPF and PF; and fiber fracture, pull-out, and matrix breakage were determined to be the main failure mechanisms in the TPF/PF composite. The rough surface of the fibers created mechanical interlocking with the matrix, resulting in enhanced mechanical characteristics. Nevertheless, a waxy layer was identified on the fiber’s surface, reducing its adhesion to the matrix and causing gaps between the fiber and matrix. Chemical treatment of the fibers would improve this issue.

## Figures and Tables

**Table 1:** Chemical composition of PF.

Component	Percent (%)
Ash	3.50 ± 0.32
Extractives	2.28 ± 0.86
Lignin	13.17 ± 1.25
Cellulose ^a^	81.05
α-cellulose	69.09 ± 0.36
β-cellulose	0.40 ± 0.25
γ-cellulose	11.40 ± 0.75

^a^ Calculated from the difference of total constituents. Values are given as mean ± standard deviation.

**Table 2:** Chemical composition of plantain fibers compared to other cellulose-based natural fibers [32,34,38,39].

Fiber	Lignin (%)	Cellulose (%)
Jute	12–13	61–71
Hemp	3	68
Sisal	8–10	65–78
Flax	3	71–81
Piassava	48.4	31.6
Bamboo	21–31	26–60
Caraua	7.5	81
Oil palm	29	40–50
Coir	40–45	32–43
Bagasse	25.3	52
Plantain (Present study)	13.2	81.1

**Table 3:** Average density and moisture content of the TPF, PF, and TPF/PF.

Property	TPF	PF	TPF/PF
Moisture (%)	10.42 ± 0.01	8.39 ± 0.72	9.90 ± 0.01
Density (g/cm^3^)	1.14 ± 0.04	0.83 ± 0.05	1.10 ± 0.06

Values are given as mean ± standard deviation.

**Table 4:** Average mechanical properties of TPF, PF, and TPF/PF.

Property	TPF	PF	TPF/PF
Tensile strength (MPa)	2.36 ± 0.21	440.30 ± 146.95	8.16 ± 1.66
Elongation (%)	24.53 ± 2.82	2.94 ± 0.77	4.09 ± 0.53
Young’s modulus (MPa)	23.62 ± 3.10	15,668.81 ± 5534.94	281.34 ± 38.58
Specific strength (MPa/g cm^3^)	2.07 ± 0.21	530.12 ± 146.95	10.74 ± 1.66
Specific modulus (MPa/g cm^3^)	20.64 ± 2.82	18,878.08 ± 5534.94	370.18 ± 38.58

Values are given as mean ± standard deviation.

**Table 5:** Average mechanical properties of various starch-based polymeric films [32,62,63,64,65,66].

Source	Tensile Strength (MPa)	Young’s Modulus (MPa)	Elongation at Break (%)
Corn starch	1.23 ± 0.07	2.31 ± 0.06	53.13 ± 0.81
Batata starch	3.51 ± 0.16	25.09 ± 2.66	70.74 ± 5.67
Rice starch	3.70 ± 0.92	9.33 ± 0.47	39.50 ± 0.92
Banana flour	1.00 ± 0.10	2.70 ± 0.70	49 ± 2.00
Banana/beet flour	1.14 ± 0.03	3.3 ± 0.10	53 ± 1.00
Chemically modified starch	12.8	1169	8.3
Commercial starch	7.90 ± 0.07	73.10 ± 5.78	33.40 ± 0.41
TPF (Present study)	2.36 ± 0.21	23.62 ± 3.10	24.53 ± 2.82

Values are given as mean ± standard deviation.

**Table 6:** Average mechanical properties of various natural fibers commonly used as composite reinforcements [32,35,67,68,69,70].

Fiber	Tensile Strength (MPa)	Young’s Modulus (GPa)	Elongation at Break (%)
Jute	187–773	13–26.5	1.5–3.1
Hemp	580–1110	70	1.6–4.5
Sisal	507–885	9.4–22	1.9–3
Flax	343–1035	27.6	1.2–3
Date	58–203	2–7.5	5–10
Bagasse	20–290	N/A	1–3
PF (Present study)	211–599	10–21	2.2–3.8

## Data Availability

The data are available from the corresponding author upon request.
